# Multidisciplinary review of teaching and assessing competency attainment by non-medical prescribers

**DOI:** 10.1186/1757-1146-8-S2-O3

**Published:** 2015-09-22

**Authors:** Paul J Bennett, Lynda Cardiff, Lisa Nissen, Anthony Short, Lauren Kunde

**Affiliations:** 1 School of Clinical Science, Queensland University of Technology, Brisbane, Qld, 4061, Australia; 2 The Podiatry Practice, Taylor Medical Centre & Mater Adults and Children's Hospital, Woolloongabba, Qld, Brisbane, 4101, Australia; 3 Queensland skin and cancer foundation, Queensland Institute of Dermatology, Holland Park, Brisbane, Australia 4121, Australia

## Background

Prescribing is a complex task, requiring specific knowledge and skills, and the execution of effective, context-specific clinical reasoning. Systematic reviews indicate medical prescribing errors have a median rate of 7% [IQR 2%-14%] of medication orders [1-3]. For podiatrists pursuing prescribing rights, a clear need exists to ensure practitioners develop a well-defined set of prescribing skills, which will contribute to competent, safe and appropriate practice.

## Aim

To investigate the methods employed to teach and assess the principles of effective prescribing in the undergraduate podiatry program and compare and contrast these findings with four other non-medical professions who undertake prescribing after training at Queensland University of Technology.

## Method

The NPS National Prescribing Competency Standards were employed as the prescribing standard. A curriculum mapping exercise was undertaken to determine whether the prescribing principles articulated in the competency standards were addressed by each profession.

## Results

A range of methods are currently utilised to teach prescribing across disciplines. Application of prescribing competencies to the context of each profession appears to influence the teaching methods used. Most competencies were taught using a multimodal format, including interactive lectures, self-directed learning, tutorial sessions and clinical placement. In particular clinical training was identified as the most consistent form of educating safe prescribers across all five disciplines. Assessment of prescribing competency utilised multiple techniques including written and oral examinations and research tasks, case studies, objective structured clinical examination exercises and the assessment of clinical practice. Effective and reliable assessment of prescribing undertaken by students in diverse settings remains challenging e.g. that occurring in the clinical practice environment.

## Conclusion

Recommendations were made to refine curricula and to promote efficient cross-discipline teaching by staff from the disciplines of podiatry, pharmacy, nurse practitioner, optometry and paramedic science. Students now experience a sophisticated level of multidisciplinary learning in the clinical setting which integrates the expertise and skills of experience prescribers combined with innovative information technology platforms (CCTV and live patient assessments). Further work is required to establish a practical, effective approach to the assessment of prescribing competence especially between the university and clinical settings.
